# The Global Role of Kidney Transplantation for the World Kidney Day Steering Committee 2012 

**Published:** 2012-02-01

**Authors:** G. Garcia-Garcia, P. Harden, J. Chapman

**Affiliations:** 1 *Nephrology Service, Hospital Civil de Guadalajara, University of Guadalajara Health Sciences Center (CUCS) Hospital 278, Guadalajara, Jal. 44280, Mexico*; 2 *Oxford Kidney Unit and Oxford Transplant Center, Churchill Hospital, Oxford, United Kingdom*; 3 *Center for Transplant and Renal Research, Westmead Millennium Institute, Sydney University, Westmead Hospital, Sydney, NSW, 2145, Australia `*

**Keywords:** Kidney, Transplantation, Renal Insufficiency, Chronic

## Abstract

World Kidney Day on March 8th, 2012, provides a chance to reflect on the success of kidney transplantation as a therapy for end-stage kidney disease that surpasses dialysis treatments, both for the quality and quantity of life, that it provides and for its cost effectiveness. Anything that is both cheaper and better, but is not actually the dominant therapy, must have other drawbacks that prevent replacement of all dialysis treatment by transplantation. The barriers to universal transplantation as the therapy for end-stage kidney disease include the economic limitations which, in some countries place transplantation, appropriately, at a lower priority than public health fundamentals such as clean water, sanitation and vaccination. Even in high-income countries the technical challenges of surgery and the consequences of immunosuppression restrict the number of suitable recipients, but the major finite restrictions on kidney transplantation rates are the shortage of donated organs and the limited medical, surgical and nursing workforces with the required expertise. These problems have solutions which involve the full range of societal, professional, governmental and political environments. World Kidney Day is a call to deliver transplantation therapy to the one million people a year who have a right to benefit.

## INTRODUCTION

Kidney transplantation is acknowledged as a major advance of modern medicine which provides high-quality life years to patients with irreversible kidney failure (end-stage renal disease, ESRD) worldwide. What was an experimental, risky and very limited treatment option fifty years ago, is now routine clinical practice in more than 80 countries. What was once limited to a few individuals in a small number of leading academic centers in high income economies, is now transforming lives as a routine procedure in most middle and high-income countries—but can do much more. The largest numbers of transplants are performed in the USA, China, Brazil and India, while the greatest population access to transplantation is in Austria, USA, Croatia, Norway, Portugal and Spain. There are still many limitations in access to transplantation across the globe. World Kidney Day on March 8th, 2012, will bring focus to the tremendous life-changing potential of kidney transplantation as a challenge to politicians, corporations, charitable organizations and health care professionals. This commentary raises awareness of the progressive success of organ transplantation, highlight concerns about restricted community access and human organ trafficking and commercialism, while also exploring the real potential for transforming kidney transplantation into the routine treatment option for ESRD across the world.

Outcomes of Kidney Transplantation 

The first successful organ transplantation is widely acknowledged to be a kidney transplant between identical twins performed in Boston on December 23rd, 1954, which heralded the start of a new era for patients with ESRD [1].

In the development years between 1965 and 1980, patient survival progressively improved towards 90% and graft survival rose from less than 50% at one year to at least 60% after a first deceased donor kidney transplant, based on immunosuppression with azathioprine and prednisolone. The introduction of cyclosporine in the mid-1980s was a major advance, leading to one-year survival rates of more than 90% and graft survival of 80% [2]. In the last 20 years, better understanding of the benefits of combined immunosuppressant drugs coupled with improved organ matching and preservation, as well as chemoprophylaxis of opportunistic infections, have all contributed to a progressive improvement in clinical outcomes. Unsensitized recipients of first deceased donor kidney transplants and living donor recipients can now expect one-year patient and transplant survival to be at least 95% and 90%, respectively [1]. New developments have led several groups to report excellent results even from carefully selected ABO blood group incompatible transplants in recipients with low titer ABO-antibodies [3]. Even for those with high titers of donor specific HLA-antibodies, who were previously “untransplantable,” better de-sensitization protocols [4] and paired kidney exchange programs [5] now afford real opportunities for successful transplantation.

Ethnic minorities and disadvantaged populations continue to suffer worse outcomes; Aboriginal Canadians, for example, have lower 10-year patient (50% *vs*. 75%) and graft (26% *vs*. 47%) survival compared with white patients [6]. African American kidney transplant recipients have shorter graft survival compared to Asian, Hispanic, and White populations in the USA [7]. In New Zealand, Maori and Pacific Island recipients of deceased donor transplants have a 50% 8-year graft survival compared to 14 years for non-indigenous recipients, in part due to differences inmortality [8]. By contrast, despite a resource poor environment, Rizvi, *et al*, report 1- and 5-year survival rates of 92% and 85%, respectively, among 2,249 living related kidney transplants in Pakistan [9], whilst in Mexico, 90% and 80% one-year survival for living and deceased donor kidney transplants, was reported among 1,356 transplants performed at a single center [10]. But, while it is possible to achieve such excellent long-term results, most patients and their families in resource poor environments are not be able to afford the high cost immunosuppressants and antiviral medications needed to reduce the risk of graft loss and mortality [11].

The Place of Kidney Transplantation in Treatment for ESRD 

Kidney transplantation improves long-term survival compared to maintenance dialysis. In 46,164 patients on the transplant waiting list in the USA between 1991 and 1997, mortality was 68% lower for transplant recipients than for those remaining on the transplant waiting list after >3 yrs follow-up [12]. The transplanted 20–39-year-old patients of both sexes were predicted to live 17 years longer than those remaining on the transplant waiting list, an effect that was even more marked in diabetics. 

The number of people known to have ESRD worldwide is growing rapidly, as a result of improved diagnostic capabilities and also the global epidemic of type 2 diabetes and other causes of chronic kidney disease (CKD). Dialysis costs are expensive even for developed countries, but prohibitive for many emerging economies. The majority of patients commencing dialysis for ESRD in low-income countries die or stop treatment within the first three months of initiating dialysis due to cost restraints [13]. The cost of maintenance hemodialysis varies considerably by country and health care system. In Pakistan, maintenance hemodialysis is reported to be US$ 1680 per year, which is beyond the reach of most of the population without humanitarian financial aid [14]. Despite exemplars, both provision of hemodialysis facilities and uptake of peritoneal dialysis remain very limited in low- and middle-income countries. Whilst the costs of transplantation exceed those of maintenance dialysis in the first year post-transplantation (*e.g.*, in Pakistan US$ 5245 *vs*. US$ 1680 in the first year), the costs are much reduced compared to dialysis in subsequent years, especially with the advent of inexpensive generic immunosuppression [15]. Transplantation thus expands access and reduces overall costs for successful treatment of ESRD.

Pre-emptive transplantation is an attractive option for both patients and payers with both reduced costs and improved graft survival [16]. Pre-emptive transplantation is associated with a 25% reduction in transplant failure and 16% reduction in mortality compared to recipients receiving a transplant after starting dialysis [17].

Transplantation of the kidney, when properly applied, is thus the treatment of choice for patients with ESRD because of lower costs and better outcomes.

Global Disparities in Access to Kidney Transplantation 

**Figure 1:**
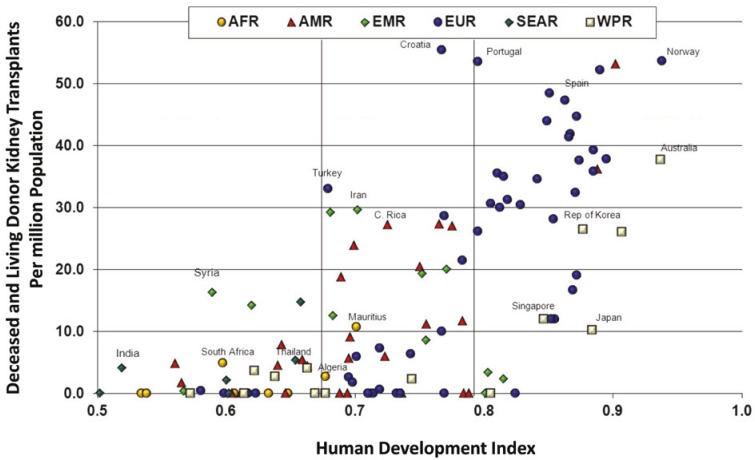
Number of deceased and living donor kidney transplants in World Health Organization member states in 2010, correlated with Human Development Index. Grouped by WHO Regions (AFR = Africa, AMR = Americas, EMR = Eastern Mediterranean, EUR = Europe, SEAR = South Eastern Asia, WPR = Western Pacific)

Substantial disparities in access to transplantation across the world are demonstrated in Figure 1 (derived from the World Health Organization/Organization Mondiale de la Sante (WHO/OMS) Global Observatory on Donation and Transplantation [18]) which demonstrates the relationship between transplant rate and Human Development Index (HDI). There is a reduced transplant rate in low- and middle-HDI countries, and a large spread of transplant rates even amongst the richer nations. Transplant rates of more than 30 per million population (pmp) in 2010 were restricted to Western Europe, USA, and Australia, with a slightly broader spread of countries achieving between 20 and 30 pmp. Iran remains different to the rest of the world through the government sponsored and patient supplemented purchase of kidneys from live donors. The overall rate of kidney transplantation exceeds that which might be predicted from the HDI alone and thus the national experiment remains of great interest outside the country.

There are also within-country disparities in transplant rates among minorities and other disadvantaged populations. In Canada, all minority groups have significantly lower transplant rates; compared to whites, rates in Aboriginal and African Canadians, Indo-Asians, and East Asians were 46%, 34%, and 31% lower, respectively [19]. In the US, transplantation rates are significantly lower among African Americans, women, and the poor, compared to Caucasians, men and the more affluent populations [20]. The situation is similar in Australia where Aboriginal Australians fare worse than non-indigenous Australians (12% *vs*. 45%) and in New Zealand where Maori/Pacific Islanders are disadvantaged (14% *vs*. 53%) [21]. In Mexico, the transplant rate among uninsured patients is 7 pmp compared with 72 pmp among those with health insurance [22].

Multiple immunologic and non-immunologic factors contribute to social, cultural, and economic disparities in transplant outcomes, including biological, immune, genetic, metabolic, and pharmacological factors as well as associated co-morbidities, time on dialysis, donor and organ characteristics, patient socio-economic status, medication adherence, access to care, and public health policies [23]. Developing countries often have especially poor transplant rates not only because of these multiple interacting factors, but also because of inferior infrastructure and an insufficient trained workforce. Deceased donation rates may also be impacted by lack of a legal framework governing brain death and by religious, cultural and social constraints. When these factors are all compounded by patient anxieties about the success of transplantation, physician bias, commercial incentives favoring dialysis and geographical remoteness, poor access to transplantation is almost inevitable for most of the world’s population. 

Improving Access to Transplantation 

Both living donation and deceased donor donation are now recognized by the WHO as critical to the capacity of nations to develop self-sufficiency for organ transplantation [24]. No country in the world, however, generates sufficient organs from these sources to meet the needs of their citizens. Austria, USA, Croatia, Norway, Portugal and Spain stand out as countries with high rates of deceased organ donors, and most developed countries are trying to emulate their success. A return to “donation after cardiac death” instead of the now standard “donation after brain death,” has enhanced the deceased organ donation numbers in several countries, with 2.8 DCD donors pmp in the US and 1.1 pmp in Australia now emanating from this source. Protocols for rapid cooling and urgent retrieval of kidneys after cardiac death, and in some circumstances other organs, have developed over the past five years to reduce the duration and consequences of warm ischemia [25]. Another strategy for increasing the rate of transplantation has been to extend the acceptance criteria for deceased organ donors. Such “extended criteria” donors require additional consideration and specific consent by the recipient. There is risk in accepting an “extended criteria” kidney since the transplants are less successful in the long term, but also a risk to waiting longer on dialysis for a standard criteria donor.

A number of strategies have been designed and implemented to reduce disparities among disadvantaged populations. The Transplantation Society has established the Global Alliance for Transplantation in an effort to reduce worldwide disparities in transplantation. The program includes collecting global information, expanding education about transplantation, and developing guidelines for organ donation and transplantation. The International Society of Nephrology (ISN) Global Outreach program has catalyzed the development of kidney transplant programs across a large number of countries with targeted fellowship training and creation of long term institutional links between developed and developing transplant centers through its Sister Center Program. This has led to the establishment of successful kidney transplantation in countries such as Armenia, Ghana and Nigeria where none existed before and expansion of existing programs in Belarus, Lithuania and Tunisia. 

A model of collaboration for dialysis and transplantation between government and the community in the resource-poor world has been successfully established in Pakistan with government assistance for infrastructure, utilities, equipment, and up to 50% of the operating budget, while the community, including affluent individuals, corporations and the public, donate the remainder [14]. In 2001, in Central America, a specialized unit of pediatric nephrology and urology was opened in Nicaragua with funds provided initially by the Associazione per il Bambino Nefropatico, a kidney foundation based in Milan, Italy supplemented by a consortium of private and public organizations, including the International Pediatric Nephrology Association and the Nicaraguan Ministry of Health. Subsequently, the Nicaraguan government and a local kidney foundation recognized the success of the program and accepted gradual transfer of the costs of treatment, including the provision of immunosuppressive medications for renal transplantation. A similar successful partnership between government and private sector has recently been reported in India [26].

There are tremendous opportunities to correct disparities in kidney disease and transplantation worldwide, but it is important to recognize that funding of ESRD treatment should be associated with funding for early detection and prevention of the progressive kidney diseases that lead to ESRD. Comprehensive programs should include community screening and prevention of CKD, especially in high-risk populations, as well as dialysis and transplantation for ESRD.

An integrated approach to the expansion of transplantation requires training programs for nephrologists, transplant surgeons, nursing staff, and donor coordinators; nationally funded organ procurement organizations providing transparent and equitable retrieval and allocation; and the establishment of national ESRD registries. 

Ethical Challenges and the Legal Environment 

The impact of the global organ donor shortage and the dramatic disparities demonstrated by the WHO data, are experienced in many different ways requiring varied responses. But one common factor is the relative wealth of the nation and the individual. The poor receive the fewest transplants and the rich are most often transplanted either in their own country or through finding an organ through illegal purchase from the poor or an executed prisoner. Trafficking in human organs and commercialization of the beneficial act of organ donation were unusual and extremely hazardous in the 1980’s, became frequent but still very hazardous in the 1990’s, then becoming a gruesomely burgeoning trade from the turn of the century. The WHO has estimated that up to 10% of all organ transplants were of commercial origin by 2005 [27]. 

The first WHO Guiding Principles in this field were agreed in 1991 and made clear by the decision of national governments to ban commercialization of organ donation and transplantation [28]. This principle was reaffirmed unanimously by the World Health Assembly in 2010 when the updated WHO Guiding Principles for human organ and tissue donation and transplantation, were endorsed [29]. Almost all countries with transplantation programs and even some without active programs have carried that ban on commercialism through to their own legislation, making it illegal to buy or sell organs. Sadly this has not prevented continuation of the trade illegally in countries such as China and Pakistan, nor has it prevented new entrants to this lucrative trade from taking advantage of their own or other nations’ impoverished and vulnerable populations to provide kidneys and even livers for the desperate wealthy in need of transplantation. 

Iran, alone, claims to have resolved national self-sufficiency for kidney transplantation through a scheme of part government, part patient-funded sale of kidneys by vendors. The resultant slow development of deceased organ donation in Iran restricting liver, heart and lung transplant programs, as well as the disparity of socioeconomic status between donors and recipients, both testify to the universality of the problems that arise from organ transplant commercialization. The restriction of transplantation to Iranian nationals only under this program has however largely ensured that this national experiment has not flowed on to create commercial organ trafficking across Iranian national borders.

The Transplantation Society and the ISN have taken a joint stand against the despoiling of transplantation therapy and victimization of the poor and vulnerable by doctors and other providers operating in these illegal programs. In 2008, more than 150 representatives from across the world from different disciplines of health care, national policy development, law and ethics came together in Istanbul to discuss and define professional principles and standards for organ transplantation. The resultant Declaration of Istanbul [30] has now been endorsed by more than 110 professional and governmental organizations and implemented by many of these organizations with a goal to eliminate transplant tourism and enhance the ethical practice of transplantation globally [31]. 

## SUMMARY

There remain major challenges to providing optimal treatment for ESRD worldwide and a need, particularly in low-income economies, to mandate more focus on community screening and implementation of simple measures to minimize progression of CKD. The recent designation of renal disease as an important non-communicable disease at the UN High Level Meeting on NCDs is one step in this direction [32]. But early detection and prevention programs will never prevent ESRD in everyone with CKD, and kidney transplantation is an essential, viable, cost-effective and life-saving therapy which should be equally available to all people in need. It may be the only tenable long-term treatment option for ESRD in low-income countries since it is both cheaper and provides a better outcome for patients than other treatment for ESRD. However, the success of transplantation has not been delivered evenly across the world, and substantial disparities still exist in access to transplantation, we remain troubled by commercialization of living donor transplantation and exploitation of vulnerable populations for profit. 

There are solutions available. These include demonstrably successful models of kidney transplant programs in many developing countries; growing availability of less expensive generic immunosuppressive agents; improved clinical training opportunities; governmental and professional guidelines legislating prohibition of commercialization and defining professional standards of ethical practice; and a framework for each nation to develop self-sufficiency in organ transplantation through focus on both living donation and especially nationally managed deceased organ donation programs. The ISN and TTS have pledged to work together in coordinated joint global outreach programs to help establish and grow appropriate kidney transplant programs in low- and middle-income countries utilizing their considerable joint expertise. World Kidney Day 2012 provides a focus to help spread this message to governments, all health authorities and communities across the world.
